# NAFLD is related to Post‐prandial Triglyceride‐enrichment of HDL Particles in Association with Endothelial and HDL Dysfunction

**DOI:** 10.1111/liv.14597

**Published:** 2020-07-25

**Authors:** Bart J. Verwer, Peter G. Scheffer, Rick P. Vermue, Petra J. Pouwels, Michaela Diamant, Maarten E. Tushuizen

**Affiliations:** ^1^ Department of Gastroenterology and Hepatology Leiden University Medical Center Leiden The Netherlands; ^2^ Department of Clinical Chemistry Amsterdam University Medical Centre Amsterdam The Netherlands; ^3^ Department of Physics & Medical Technology Amsterdam University Medical Centre Amsterdam The Netherlands; ^4^ Department of Internal Medicine Amsterdam University Medical Centre Amsterdam The Netherlands

**Keywords:** HDL, metabolic syndrome, NAFLD ‐ post‐prandial dyslipidemia ‐ type 2 diabetes

## Abstract

NAFLD is closely related with the metabolic syndrome (MetS) and increased risk of cardiovascular disease. Liver fat associates with post‐prandial hypertriglyceridemia, potentially contributing to triglyceride‐enrichment of high‐density lipoproteins (HDL‐TG), and subsequent HDL dysfunction. We assessed liver fat by MR spectroscopy, and its association with HDL physiochemical properties, and endothelial function, measured as flow‐mediated dilation (FMD), before and following three consecutive meals, in 36 men with type 2 diabetes mellitus (T2DM), with the MetS, and controls. Plasma triglycerides increased significantly following the meals (*P *< .001). Fasting HDL‐TG was highest in T2DM, relative to MetS and controls (*P* = .002), and increased post‐prandially in all groups (*P *< .001). HDL function was negatively associated with HDL‐TG following three meals (*r* = −.32, *P*<.05). Liver fat associated with HDL‐TG after three meals (*r* = .65, *P *< .001). HDL‐TG was independently associated with FMD following three consecutive meals (*r* = −.477, *P* = .003). We conclude liver fat is associated with post‐prandial HDL‐TG enrichment which was closely related with endothelial and HDL dysfunction.

**Abbreviations**
CETP: cholesteryl ester transfer protein
CVD: Cardiovascular disease
FMD: flow‐mediated dilatation
LPL: lipoprotein lipase
MetS: metabolic syndrome
T2DM: type 2 diabetes mellitus
TG: triglycerides

## 1 BACKGROUND

Non‐alcoholic fatty liver disease (NAFLD) is common in type 2 diabetes mellitus (T2DM), and closely related with features of the metabolic syndrome (MetS) and with an increased risk of cardiovascular disease (CVD).^1^, ^2^ The pathogenesis of NAFLD is complex and involves multiple factors and pathways which constitute vicious circles, yet centrally stands insulin resistance. The pro‐atherogenic lipid profile in subjects with NAFLD is characterized by elevated levels of fasting and post‐prandial triglycerides, low HDL cholesterol, and an increase in small dense LDL particles.^3^ In hypertriglyceridemic states, the net transfer of triglycerides from triglyceride‐rich lipoproteins (VLDLs and chylomicrons) to HDL particles, as mediated by cholesteryl ester transfer protein (CETP),^4^ is enhanced, yielding (the formation of) large, triglyceride‐rich, cholesterol ester‐core‐depleted HDL particles.^5^, ^6^ These HDL particles are the preferred substrate for the enzyme hepatic lipase, which hydrolyzes HDL triglycerides and promotes hepatic HDL uptake.

Liver fat content has been shown to be associated with alterations of the anti‐atherogenic HDL subfractions, but not suggest a direct causal link per se.^5^, ^7^ There is evidence that functionally defective HDL is a cause of increased cardiovascular risk in NAFLD patients.^8^, ^9^ The association of post‐prandial triglyceride elevations and impaired vascular endothelial function has also been established.^10^ Previous studies showed that triglyceride‐enrichment of HDL may alter anti‐atherogenic capacities of this lipoprotein class, including anti‐oxidative and anti‐inflammatory properties, adversely affecting the ability of HDL to protect the endothelium and vascular reactivity.^11^, ^12^ However, the independent association of liver fat content and post‐prandial HDL compositional and functional changes has hitherto not been addressed.

In the present study, we first investigated whether liver fat content is associated with post‐prandial altered HDL composition, especially triglyceride content, and its anti‐oxidative function.^13^ Second, we assessed the interrelationship of liver fat, physicochemical properties of HDL particles and endothelial function in vivo, measured as flow mediated dilatation (FMD), following three consecutive meals during a 16 hours period in males with T2DM, males with the MetS and healthy controls.

## 2 METHODS

### 2.1 Subjects

Caucasian males, aged 40‐65 years, with T2DM (n = 12) or with the MetS (n = 12), and 12 age‐matched healthy males were recruited by advertisement and studied after obtaining written informed consent. Diet, sulphonylurea and/or metformin were the only glucose‐lowering treatments allowed in the type 2 diabetic group. To disentangle the possible role of hyperglycaemia, males with the MetS had to meet three of five inclusion criteria based on NCEP/ATP III criteria, without having hyperglycaemia during a 75‐g oral glucose tolerance test. Healthy control males were overweight (BMI >25 kg/m^2^) without any other components of the MetS. Claustrophobia, excess alcohol intake (>20 units/wk), history of hepatitis and/or pancreatitis, abnormal liver and renal function tests (>2 times upper limits of normal), recent (<3 months) changes in weight (≥5%) and/or medication, history or current use of glucocorticosteroids, lipid‐lowering drugs (including statins and fibrates), insulin and/or thiazolidinediones, were exclusion criteria. Participants were instructed to omit their medication during the examination and to refrain from heavy physical activities during the previous 24h. The local ethics committee approved the study and the investigation conformed to the principles outlined in the Declaration of Helsinki.

### 2.2 Study design

After an overnight fast, participants were admitted in the research unit for a 16h period and received three consecutive, isocaloric (900 kcal) meals (75 g carbohydrates, 50 g fat (60% saturated), 35 g protein), at time points 9.00 AM, 1.00 PM and 5.00 PM. Breakfast consisted of an EggMcMuffin®, croissant with butter and marmalade, 200 mL of milk, combined with 20 mL of cream, and 13 mL of syrup. The lunch consisted of a Quarterpounder®, croissant with butter, 200 mL of milk and 16 mL of syrup. Diner consisted of a Quarterpounder®, 90 gr of French fries, 175 gr of salad and 200 mL of water. The subjects were instructed to consume each meal within 15 minutes. Blood samples were drawn before and 2, 4, 6, 8, 12 and 16h after breakfast. To avoid lipoprotein lipase (LPL) activation by physical activity, participants remained in the semi‐recumbent position during the whole testing day.

### 2.3 Biochemical measurements

Plasma glucose concentrations were measured by hexokinase‐based technique (Roche diagnostics, Mannheim, Germany) and insulin concentrations by immunoradiometric assay (Centaur, Mijdrecht, The Netherlands). Plasma total cholesterol, HDL cholesterol and triglycerides were determined by enzymatic methods (Modular, Hitachi, Japan). LDL cholesterol was calculated by the Friedewald formula. Plasma apoB‐lipoprotein was measured by immunochemical methods (Thermo Electron Ov, Vantaa, Finland). Glycated haemoglobin was measured with cation exchange chromatography (Menarini Diagnostics, Florence, Italy). High‐sensitive C‐reactive protein (hs‐CRP; mg/L) was measured in duplicate using ELISA (Sanguin, Amsterdam, The Netherlands). Insulin resistance was estimated according to the homoeostasis model assessment of insulin resistance [HOMA‐IR; (plasma glucose x insulin)/22.5].

### 2.4 HDL composition and function

HDL was isolated from EDTA plasma by ultracentrifugation for measurement of triglyceride, cholesterol and protein content. HDL triglyceride and HDL cholesterol content was expressed as nmol per gram protein (nmol/g). HDL‐TG was calculated as the HDL triglyceride: total cholesterol ratio and expressed as nmol/nmol. The anti‐oxidative function of HDL was assessed by monitoring its capacity to inhibit the oxidation of dichlorodihydrofluorescein (Invitrogen) by oxidized 1‐palmitoyl‐2‐arachidonoyl‐phosphathidylcholine (Avanti Lipids).^9^ Briefly, in a total volume of 790 µL phosphate‐buffered saline (pH 7.4), 35 µL of a normal LDL solution (final concentration of 18 µmol/L cholesterol), 35 µL of test HDL cholesterol (final concentration of 11 µmol/L cholesterol), 20 µL oxidized 1‐palmitoyl‐2‐arachidonoyl‐sn‐phosphatidylcholine (final concentration 50 µmol/L), and 10 µL of dichlorofluorescein solution (final concentration of 40 µmol/L) were incubated in glass tubes for 2 hours at 37°C. Afterwards the fluorescence intensity was determined with a HTS 7000 plate reader (Perkin Elmer) at 485 and 530 nm (excitation and emission wavelength respectively). The intra‐assay CV was 3.4%.

### 2.5 Endothelial function

Before each blood collection, FMD was measured at the right brachial artery by a single observer using ultrasound (Wall‐track System, PieMedical, Maastricht, The Netherlands), as previously described.^10^ FMD refers to dilation of an artery following induced increased shear stress and release of nitric oxide by the endothelium, reflecting one of its functions.

### 2.6 Liver fat content

Using a 1.5‐T whole‐body system MRI (Sonata; Siemens, Erlangen, Germany), liver fat content was measured after an overnight fast on a separate occasion within 2 weeks, at three positions in the liver and calculated by user‐independent spectral quantification as previously described in detail.^14^

### 2.7 Statistical analysis

Results are presented as the mean ± SE or medians (interquartile range). Sixteen hour area under the curve (AUC_0‐16h_) were calculated according to the trapezoid rule. Differences between groups were calculated using ANOVA and post‐hoc analyses (Bonferroni). Non‐normally distributed data were log transformed. The associations of liver fat content, FMD and HDL cholesterol composition and function were assessed by univariate and multivariate linear regression analyses. A value of *P* < .05 was considered statistically significant.

## 3 RESULTS

The baseline characteristics of the T2DM, MetS and healthy groups are listed in Table 1. From the T2DM males, four were using diet only, six metformin only, one sulphonylurea only and one the combination as glucose‐lowering therapy.

**Table1 1:** Baseline characteristics of the study population

	T2DM		MetS	Controls	*P*
N	12		12	12	—
Age (years)	54.6 ± 1.0		57.2 ± 1.8	55.3 ± 2.2	.556
Body Mass Index (kg/m^2^)	32.6 ± 1.3		30.6 ± 1.0	27.1 ± 0.8	.002
Waist Circumference (cm)	111.8 ± 2.9		110.9 ± 2.9	100.7 ± 2.5	.012
Systolic Blood Pressure (mm Hg)	137 ± 4		140 ± 4	121 ± 2	.001
Diastolic Blood Pressure (mm Hg)		83 ± 1	84 ± 2	74 ± 1	<.001
HbA1c (%)	7.2 ± 0.3		5.9 ± 0.1	5.6 ± 0.1	<.001
Fasting Plasma Glucose (mmol/L)	8.9 ± 0.7		5.6 ± 0.1	5.4 ± 0.1	<.001
Post 75 g OGTT glucose (mmol/L)	15.6 ± 1.2		6.2 ± 0.2	5.0 ± 0.3	<.001
HOMA‐IR	3.9 ± 0.6		2.1 ± 0.3	1.1 ± 0.2	<.001
hs‐CRP (mg/L)	1.8 ± 0.4		2.0 ± 0.5	0.8 ± 0.2	.140
ALAT (U/L)	36 ± 3.8		31 ± 3.8	28 ± 4.8	.417
Gamma‐GT (U/L)	35 ± 4.2		30 ± 3.2	23 ± 3.5	.109
Total Cholesterol (mmol/L)	5.1 ± 0.2		5.4 ± 0.3	5.1 ± 0.3	.534
LDL‐C (mmol/L)	3.0 ± 0.5		3.3 ± 0.8	3.1 ± 0.9	.643
Triglycerides (mmol/L)	2.2 ± 0.4		2.2 ± 0.2	1.0 ± 0.1	.002
HDL‐C (mmol/L)	1.0 ± 0.1		1.1 ± 0.1	1.5 ± 0.1	.001
Fasting HDL‐TG (mmol/mmol)	148 ± 16		108 ± 13	78 ± 8	.002
Fasting FMD (%)	4.9 ± 0.5		5.7 ± 0.7	7.8 ± 0.5	.003
Liver Fat Content (%)	17.8 (9.4‐39.0)		9.2 (3.4‐11.5)	3.4 (1.8‐9.3)	.001

Values are means ± SE or median (interquartile range). *P* value is calculated by ANOVA. T2DM, type 2 diabetes mellitus; MetS, metabolic syndrome; BMI, body mass index; OGTT, oral glucose tolerance test; HOMA‐IR, homeostasis model assessment of insulin resistance; hs‐CRP, high‐sensitive C‐reactive protein; LDL‐C, LDL cholesterol; HDL‐C, HDL cholesterol; FMD, flow‐mediated dilatation.

**Figure 1:**
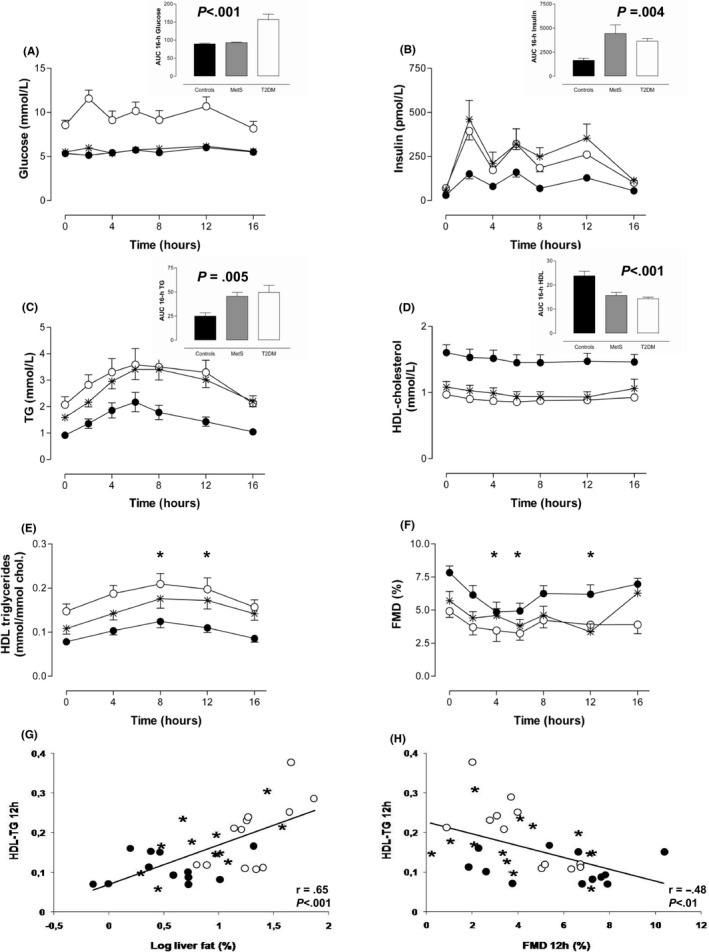
The 16‐h course of plasma glucose (A), triglyceride (B), apoB (C), HDL cholesterol (D), HDL‐TG concentrations (E), and flow‐mediated dilatation (FMD)(F) after three high‐fat mixed meals in T2DM (open circles), MetS (asterisk) and healthy males (solid circles). Bars (black, healthy males; grey, metabolic syndrome (MetS); white, type 2 diabetes mellitus (T2DM)) in the insets represent respective 16‐h AUC values. The *P* value given for 16‐h AUC difference (ANOVA). Data are mean ± SE. Scatter plots representing the relationship between liver fat content (G) and FMD (H), and HDL‐TG enrichment following three high‐fat mixed meals in the whole study population (groups are indicated by open circles (T2DM), asterisk (MetS) and solid circles (healthy males)). Pearson correlation coefficients are shown

Figure 1A‐D depicts the 16h course and AUC_0‐16h_ of the post‐prandial metabolic responses. Fasting and AUC_0‐16h_ plasma glucose concentrations were similar in both non‐diabetic groups, but differed significantly from T2DM males (*P* < .001) (Figure 1A). Plasma triglycerides increased significantly after the meals in all groups. HDL cholesterol concentrations decreased in all groups following the consecutive meals, however, AUC_0‐16h_ of HDL was significantly lower in the two dysmetabolic groups, as compared to controls. Post‐prandial HDL‐TG increased significantly in all groups, especially at time point 12 hours (ie 8 hours after breakfast and 4 hours after lunch), all *P* < .001 (Figure 1E). At baseline, there was no difference in anti‐oxidative function of HDL between the three groups. The post‐prandial decrease in anti‐oxidative capacity of HDL, as observed in T2DM, did not reach statistical significance (*P* = .12). Post‐prandial HDL function, adjusted for baseline, was negatively associated with the increase in HDL‐TG following three consecutive meals (*r* = −.32, *P* < .05).

Baseline FMD differed between groups, and was correlated with fasting HDL‐TG (*r* = −.43, *P* < .01). FMD deteriorated post‐prandially in all three groups (Figure 1F) and AUC_0‐16h_ FMD was impaired in MetS and T2DM males versus controls (*P *= .002). During the post‐prandial state, FMD at time points 8 and 12h following breakfast was negatively associated with corresponding HDL‐TG (both *r* = −.48, *P* = .003)(Figure 1H).

Liver fat content was positively associated with AUC_0‐16h_ glucose (*r* = .44, *P* < .01), triglycerides (*r* = .62, *P < *.001), apoB (*r* = .48, *P < *.01), insulin (*r* = .62, *P < *.001) and inversely with AUC_0‐16h_ HDL cholesterol (*r* = −.56, *P < *.001) and FMD (*r* = −.44, *P < *.01). As shown in Figure 1G, post‐prandial HDL‐TG following three meals was strongly associated with liver fat content (*r* = .65, *P* < .001).

Multivariate analysis was performed in the pooled groups to study the independent interrelationship of post‐prandial FMD, liver fat content, HDL‐TG, and HDL function. FMD at 12h following breakfast was entered as a dependent variable, and age, HDL‐TG, plasma triglyceride and glucose concentration at corresponding time point (*t* = 12 hours), liver fat content, HDL function at *t* = 12 hours were entered as independent variables into the model. Also, additional multivariate analyses, including BMI, age, pre‐load glucose and insulin, did not change the association. Stepwise regression analyses, revealed HDL‐TG at the corresponding time point as independently associated with post‐prandial FMD; *r* = −.477, *P* = .003 (Coefficient B −15.220 [−25.007 to −5.433]).

## 4 DISCUSSION

In the present study, we demonstrated that NAFLD is associated with HDL‐TG enrichment after three consecutive meals in men with T2DM and men with the MetS. Furthermore, post‐prandial HDL‐TG enrichment was independently related to endothelial dysfunction measured by FMD and closely associated with change in the anti‐oxidative capacity of HDL following three consecutive mixed meals.

Our findings based on physiological stimuli, confirm but rather extend previous results by Patel et al who demonstrated that infusion of an artificial fat emulsion (Intralipid) results in HDL‐TG enrichment with impaired endothelial function, as assessed by inhibition of (in vitro) endothelial cell adhesion molecule expression, in young healthy males.^12^ In addition, results by Wang and colleagues showed that post‐prandial TG‐enriched lipoproteins collected from patients with and without hypertriglyceridemia, modulate endothelial function in vivo by several pathways.^15^, ^16^ It was demonstrated that TG‐enriched lipoproteins especially aggravate endothelial function in low‐grade inflammatory states as seen in abdominal obesity and NAFLD.

Our results suggest that NAFLD is related to exaggerated and prolonged post‐prandial dysmetabolism, including HDL‐TG enrichment that independently is associated with post‐prandial endothelial dysfunction. We could only speculate whether this prolonged dysmetabolism is the result of an impaired clearance of TG‐enriched HDL by the liver, or an increased TG enrichment of HDL by, for example, elevated CETP levels.

A limitation of the present study is that we isolated and examined the total class of HDL instead of the HDL_2_ and HDL_3_ subclasses.

## 5 CONCLUSIONS

In men with T2DM and the MetS exposure to three consecutive meals produces exaggerated HDL‐TG enrichment, which was closely associated with liver fat content, and HDL and endothelial dysfunction. Our findings may link liver fat accumulation and post‐prandial dysmetabolism to the high CVD risk present in T2DM and the MetS. Future studies should elucidate whether liver fat regression by therapeutic intervention may lead to an improvement of CVD risk.

## CONFLICT OF INTERESTS

All authors have no competing financial and non‐financial interests.

## AUTHOR CONTRIBUTIONS

MET, PGS and MD were involved in study concept and design, acquisition of data, analysis and interpretation of data, critical revision of the manuscript for important intellectual content and statistical analysis. BV and PJP were involved in drafting of the manuscript, critical revision of the manuscript for important intellectual content and statistical analysis. RPV was involved in drafting of the manuscript, and administrative and technical support.
